# The Heating of the Street Cars

**Published:** 1891-01

**Authors:** 


BUFFALO MEDICAL AND SURGICAL JOURNAL

A MONTHLY REVIEW OF MEDICINE AND SURGERY.

*EDITORS:*

THOMAS LOTHROP, M. D. - - WM. WARREN POTTER, M. D.

All communications, whether of a literary or business character, should be addressed to the editors : 284 FRANKLIN STREET, BUFFALO, N. Y.

VOL. XXX. JANUARY, 1891. No. 6.

### Editorial.

### THE HEATING OF STREET CARS.

WE HAVE heretofore called attention to the desirability of warming the street cars, and have presented some of the reasons that, in our opinion, prove the advantage of so doing over the present system. It is not necessary to repeat what has been said upon former occasions, but we recur to the subject now in order to call attention to a recent editorial in *The Buffalo Courier* which expresses so well the proper position to take on this matter, that we print it entire.

It is not often that hygienic questions are discussed by the lay press without betraying the ignorance of the writer; and an important step is taken in the diffusion of knowledge when we find such correct conclusions given the wide publicity of an editorial in a leading daily as the following :

Recent remarks of *The Courier* on the heating of street cars have drawn comments from esteemed contemporaries of Rochester, a city whose cars are heated. The *Rochester Herald* approves the heating, while the *Rochester Union* disapproves. The *Union* says that "usually the air in the heated cars is vile." If by this it is meant to say that heating the air fouls it, the comment is not sound. Cold air, as well as warm, may be foul. In fact, in an unheated car, the tendency to keep the ventilators closed and breathe the air over and over, is stronger than in a heated car. It is this rebreathing, and not the heat, that vitiates the air. So far as the heat affects the air, its tendency is to create currents and promote an interchange of the air inside with that outside — and this is ventilation. The *Herald* says :

"All who had to travel some distance on the street cars in this city before the heaters were introduced, know not only that they suffered much discomfort, but frequently contracted colds by leaving the warm rooms of their homes and offices and sitting for from ten to twenty-five minutes in a car, the temperature of which was often far below the freezing point."

This has been the case in Buffalo. Those who have ridden short distances probably have not suffered much from this cause, but several of the Buffalo routes are an hour long, and that is time enough to get dangerously chilled in a cold day.

Our neighbor, the *Commercial*, says: 'There is not a more reliable disease-breeder than the atmosphere of a heated street car." Here, again, we have the mistake of assuming that hot air is necessarily less pure than cold air. This is entirely erroneous. The purity of the air and its temperature are quite distinct matters.

The truth is, that in relation to this subject, as to most, there is a golden mean. A car heated too much and a car not heated at all are equally unsuited to comfort and dangerous to health. The subject is one of considerable moment here just now. The general manager of the Buffalo street car lines has stated that the cars are to be heated, and heated with stoves. But, Winter is upon us, and no cars are yet heated. That a popular demand for heated cars has long existed here cannot be disputed. As the number of passengers increases, the interest in the subject also increases. As the heaters have evidently not yet been procured, and as the question of their form and character may be still open, it can hardly be out of the way, in the interest of the thousands of people whose comfort (and perhaps health) is involved, to urge the managers to be cautious about going from one extreme to the other. If the cars can be *warmed* without being *heated*, a good thing will be accomplished. It is a matter to which much attention has been paid, and for which many devices have been contrived. Some care and pains in examining them and securing the best will be well expended.

